# Overweight Associated with Increased Risk of Erosive Esophagitis in a Non-Obese Taiwanese Population

**DOI:** 10.1371/journal.pone.0077932

**Published:** 2013-11-01

**Authors:** Pei-Chi Chih, Yi-Ching Yang, Jin-Shang Wu, Yin-Fan Chang, Feng-Hwa Lu, Chih-Jen Chang

**Affiliations:** 1 Department of Family Medicine, Madou Sin-Lau Hospital, Tainan, Taiwan, ROC; 2 Department of Family Medicine, National Cheng Kung University Hospital, Tainan, Taiwan, ROC; 3 Department of Family Medicine, College of Medicine, National Cheng Kung University, Tainan, Taiwan, ROC; Robert Gordon University, United Kingdom

## Abstract

**Objective:**

To investigate the relationship between overweight and erosive esophagitis (EE) in a non-obese Taiwanese population.

**Design and Methods:**

A total of 7,352 subjects (non-obese, 5,826; obese, 1,526) from a health examination center at National Cheng Kung University Hospital were enrolled. Central obesity was defined by a waist circumference (WC) ≥90 cm in male and 80 cm in female. Overweight was defined as body mass index (BMI) of 24–26.9 kg/m^2^, and general obesity as BMI ≥27 kg/m^2^. The Los Angeles classification was adopted to determine the presence of EE.

**Results:**

There were significant differences in the prevalence of central obesity and different BMI status between subjects with and without EE in total and non-obese population. In total population, multivariate analyses revealed central obesity (OR, 1.17, 95% CI, 1.02–1.34, *p* = 0.021) and being obese (OR, 1.28, 95% CI, 1.07–1.52, *p* = 0.007)/overweight (OR, 1.25, 95% CI, 1.08–1.45, *p* = 0.003) had positive associations with EE in different model, respectively. When considering the joint effect of central obesity and BMI status, overweight (OR, 1.22; 95% CI, 1.04–1.44; *p* = 0.016) remained as an independent associated factor of EE but central obesity (OR, 1.06; 95% CI, 0.89–1.26; *p* = 0.549)/being obese (OR, 1.22; 95% CI, 0.98–1.53; *p* = 0.082) did not. As for non-obese group, separate model showed central obesity (OR, 1.19, 95% CI, 1.00–1.40, *p* = 0.046) and overweight (OR, 1.24; 95% CI, 1.07–1.44, *p* = 0.005) was positively associated with EE, respectively. However, being overweight (OR, 1.20; 95% CI, 1.02–1.42, *p* = 0.030) but not central obesity (OR, 1.08; 95% CI, 0.90–1.31; *p* = 0.398) was positively related to EE with considering the effect of overweight and central obesity simultaneously.

**Conclusion:**

Overweight effect on EE was more detrimental than central obesity in non-obese subjects. In addition, male gender, hiatus hernia and alcohol use were also associated with increased risk of EE.

## Introduction

Gastroesophageal reflux disease (GERD), defined as the abnormal reflux of gastric contents into the esophagus, is a chronic relapsing disease that results in not only troublesome reflux symptoms and a variety of extra-esophageal manifestations which impair quality of life, but also more serious problems, such as reflux esophagitis [1]. If such patients develop reparative erosive esophagitis (EE), then this may lead to long-term complications as Barrett’s metaplasia and esophageal adenocarcinoma [2]–[4].

The prevalence of EE in Asia shows considerable variability, ranging from <1.0% to 20.8%, depending on the different races and ethnic groups studied [5]. In recent decades, there has been a significant rise in the prevalence of EE worldwide [5], [6], and the clinical significance of the preventable risk factors of EE thus deserve more attention.

Obesity is believed to be an important factor in the emergence of EE, in addition to other well-known risk factors, such as male gender, hiatus hernia, alcohol use and smoking [2], [5]–[9]. Several recent studies from Asia find correlations between central obesity and EE, with consistently significant associations having been confirmed in a number of works [10]–[12]. However, the subjects in these studies were a mix of individuals with different BMI statuses, including normal weight, overweight and obesity, and no studies have been carried out with a non-obese population to examine the association between EE and BMI and waist circumference (WC). This study was thus carried out to investigate the relationship between central obesity/overweight and EE in a non-obese Taiwanese population.

## Materials and Methods

### Ethics Statement

This study was approved by the Institutional Review Board of the National Cheng Kung University of Medicine (B-ER-101–189). Because the participants only agreed to have their questionnaire data and related examination results analyzed anonymously, we performed the analysis based on a secondary data without personal ID.

### Study Population and Selection of Study Participants

In this cross-sectional study, the participants consisted of 11,931 subjects who voluntarily underwent upper esophagogastroduodenoscopy as a part of a routine health check-up at National Cheng Kung University Hospital in Tainan between January 2001 to August 2009. Year of check-up were classified as 2001∼2003, 2004∼2006, and 2007∼2009 three groups. There were 7,352 subjects included in the final analysis after excluding subjects with a history of esophageal or gastric cancer (n = 18), receiving current medication therapy for gastrointestinal disease (n = 679), coronary heart disease (n = 203), cardiac arrhythmia (n = 240), asthma (n = 4) and arthritis (n = 252), and incomplete data (n = 3,301). The subjects were divided into non-obese (n = 5,826) and obese group (n = 1,526).

### Questionnaire

All subjects completed a structured questionnaire which included demographic information, medical history, medication history and lifestyle habits (cigarette smoking, alcohol use, tea drinking habits and details of physical exercise). Smoking was categorized as nonsmokers and current smokers, with the latter defined as at least 20 cigarettes per month for more than half-a-year. Alcohol use was classified into none and current alcohol use, with the latter defined as at least one alcoholic drink per week for more than half-a-year. Tea drinking habit was divided into none and current tea drinking, with the latter defined as at least one cup of tea per week for more than half-a-year. Regular exercise was defined as vigorous exercise with a minimum of 20 minutes per time, three times or more per week, according to the recommendations of the American College of Sport Medicine [13].

### Anthropometric and Laboratory Measurements

Anthropometric measurements, blood pressure (BP), and blood sampling were carried out by well-trained nurses. Body weight (to the nearest 0.1 kg) and body height (to the nearest 0.1 cm) were measured using a certified machine. The body mass index (BMI), calculated as the measured weight (in kilograms) divided by the square of measured height (in meters) was categorized as follows: normal (<24 kg/m^2^), overweight (24–26.9 kg/m^2^) and obese (≥27 kg/m^2^) [14]. The WC was measured at the midway between the lower border of rib cage and iliac crest when the subjects were standing at the end of normal expiration. A BP monitor (1846SX; Johnson and Johnson, assembled in Mexico) was used to measure two readings of systolic blood pressure (SBP) and diastolic blood pressure (DBP) in a supine position after at least 10 minutes of rest.

Following a 12-hour overnight fast, blood samples were obtained from each subject, and laboratory tests included fasting plasma glucose, uric acid, creatinine, total cholesterol, triglycerides (TG) and high density lipoprotein cholesterol (HDL-C). Individuals without a medical history of diabetes received a 75-gm oral glucose tolerance test. Fasting plasma glucose (FPG) and two-hour post-load glucose were sampled and measured by a hexokinase method (Roche Diagnostic GmbH, Mannheim, Germany). Uric acid and creatinine were measured by enzymatic methods using the Hitachi 7600-110 automated chemistry analyzer (Hitachi, Tokyo, Japan). Total cholesterol, TG and HDL-C were determined in the central laboratory of National Cheng Kung University Hospital with an autoanalyzer (Hitachi 747E, Tokyo, Japan).

Central obesity was identified as a WC greater than 90 cm in males and a WC greater than 80 cm in females, according to the Asia-Pacific criteria of the World Health Organization [15]. Diabetes mellitus (DM) was determined based on the diagnostic criteria of the American Diabetes Association, including an FPG level of 6.99 mmol/L or more, a two-hour post-load glucose level of 11.1 mmol/L or more, or a positive history of DM [16]. Diagnosis of hypertension (HTN) was based on the suggestion of the seventh report of the Joint National Committee, including a mean SBP greater than 140 mmHg, a mean DBP greater than 90 mmHg, or a self-reported history of HTN [17]. According to the National Cholesterol Education Program (NCEP) Adult Treatment Panel III (ATP III) guidelines [18], hypertriglyceridemia was defined as TG levels greater than 1.70 mmol/L, and low HDL-C levels were defined as less than 1.04 mmol/L in males and less than 1.30 mmol/L in females.

### Upper Gastrointestinal Endoscopy

Upper gastrointestinal endoscopy, using LUCERA gastroscopes (GIF-XQ260, Olympus, Tokyo, Japan), was performed by experienced gastrointestinal specialists. The Los Angeles classification, proposed by the tenth World Congress of Gastroenterology in 1994 and revised in 1999, was adopted to determine the presence of EE [19]. The presence or absence of hiatus hernia was determined from the endoscopy results.

### Statistical Analysis

Demographic and clinical characteristics were compared between subjects with and without EE. Continuous numerical variables were presented as means ± standard deviation (SD) and categorical variables as number and percent. Student’s *t* test was used for the comparison of continuous variables between two groups, and the chi-squared test for the comparison of categorical variables. Since triglyceride and creatinine were skewed data, median (interquartile range) and non-parametric test were used for their comparison between two groups. Logistic regression analysis was used to examine the associations between central obesity/BMI status and EE in total, non-obese and obese subgroups. Other predictor variables included age, gender, year of check-up, creatinine, uric acid, HTN, DM, hypertriglyceridemia, low HDL-C, hiatus hernia, regular exercise, smoking, tea drinking and alcohol use. The odds ratios (OR) and 95% confidence interval (CIs) were computed. The SPSS, version 17.0 (Chicago, Illinois, USA) was used for all statistical analyses, and a *P* value of less than 0.05 was viewed as statistically significant.

## Results

A total of 7,352 subjects (non-obese, 5,826; obese, 1,526) were recruited for analysis. EE was found in 1,463 (19.9%), 1,096 (18.8%) and 367 (24%) in total population, non-obese subjects and obese subgroup, respectively. Table 1 shows the comparison of clinical characteristics between subjects with and without EE. In total population, there were significant differences in age, gender, year of check-up, BMI, different BMI statuses, WC, DBP, FPG, uric acid, creatinine, TG, HDL-C, and the prevalence of hiatus hernia, central obesity, diabetes mellitus, hypertriglyceridemia, low HDL-C, regular exercise, current tea drinking, smoking, and alcohol use between subjects with and without EE. In non-obese group, those with EE were more likely to be male and had higher values of BMI, WC, DBP, FPG, uric acid, creatinine, and TG, but a lower level of HDL-C than those without EE. There were significant differences in the prevalence of hiatus hernia, central obesity, overweight, hypertriglyceridemia, regular exercise, current tea drinking, current smoking and alcohol use between subjects with and without EE.

**Table 1:** Clinical characteristics of subjects with or without erosive esophagitis.

	Total population		Non-obese population	
	EE (+)	EE (−)	EE (+)	EE (−)
	(N = 1463)	(N = 5889)	(N = 1096)	(N = 4730)
Age (year)	48.7±11.9	49.5±12.1*	48.9±12.1	49.2±12.3
Male gender	1106 (75.6)	3356 (57.0)***	808 (73.7)	2558 (54.1)***
Body height (cm)	166.0±7.9	163.3±8.3***	165.9±7.9	163.1±8.2***
Body weight (kg)	69.7±12.4	65.1±11.9***	65.3±9.2	61.6±9.4***
BMI (kg/m^2^)	25.2±3.5	24.3±3.5***	23.7±2.2	23.1±2.4***
BMI				
≥27	367 (25.1)	1159 (19.7)***		
24–26.9	551 (37.7)	1863 (31.6)	551 (50.5)	1863 (39.4)***
<24	545 (37.3)	2867 (48.7)	541 (49.5)	2862 (60.6)
WC (cm)	86.8±10.1	83.4±10.2***	83.4±8.0	80.0±8.3***
Central obesity	657 (44.9)	2258 (38.3)***	317 (28.9)	1172 (24.8)***
SBP (mmHg)	118.9±16.4	118.5±18.0	116.9±16.0	116.5±17.7
DBP (mmHg)	71.4±10.6	69.9±11.1***	70.2±10.2	68.7±10.8***
Hypertension	322 (22.0)	1287 (21.9)	198 (18.1)	881 (18.6)
FPG (mmol/L)	5.3±1.5	5.2±1.4**	5.2±1.5	5.1±1.4*
Diabetes mellitus	225 (15.4)	787 (13.4)*	147 (13.4)	538 (11.4)
Total cholesterol (mmol/L)	5.13±0.97	5.12±0.97	5.10±0.99	5.10±0.97
TG (mmol/L)^#^	1.4 (1.0)	1.2 (0.9)***	1.3 (0.9)	1.1 (0.8)***
HDL-C (mmol/L)	1.2±0.3	1.3±0.4***	1.2±0.3	1.3±0.4***
Hypertriglyceridemia	544 (37.2)	1582 (26.9)***	342 (31.2)	1070 (22.6)***
Low HDL-C	625 (42.7)	2319 (39.4)*	414 (37.8)	1659 (35.1)
Uric acid ( µmol/L)	381.9±87.4	258.7±92.8***	371.2±84.5	346.8±89.8***
Creatinine ( µmol/L)^#^	79.6 (17.7)	79.7(26.5)***	79.56 (17.68)	70.72(26.52) ***
Current tea drinking	749 (51.2)	2743 (46.6)**	546 (49.8)	2141 (45.3)*
Current smoking	315 (21.5)	882 (15.0)***	225 (20.5)	666 (14.1)***
Current alcohol use	365 (24.9)	929 (15.8)***	250 (22.8)	697 (14.7)***
Regular exercise	195 (13.3)	657 (11.2)*	366 (33.4)	1467 (31)*
Hiatus hernia	241 (16.5)	52 (0.9)***	173 (15.8)	43 (0.9)***
Year of check-up				
2001∼2003	265 (18.1)	913 (15.5)*	196 (17.9)	719 (15.2)
2004∼2006	639 (43.7)	2703 (45.9)	479 (43.7)	2142(45.3)
2007∼2009	559 (38.2)	2273 (38.6)	421 (38.4)	1869 (39.5)

Data expressed as number (%), mean ± SD or median (IQR)^#.^

*p* value: *<0.05; **<0.01; ***<0.001.

BMI: body mass index; WC: waist circumference; SBP: systolic blood pressure; DBP: diastolic blood pressure; FPG: fasting plasma glucose; HDL-C: high density lipoprotein-cholesterol.

In the multivariate regression analyses on the risk for EE in total population (Table 2), model 1 shows that central obesity (odds ratio (OR), 1.17; 95% confidence interval (CI), 1.02–1.34; *p* = 0.021) was positively associated with EE in addition to age (OR, 0.99; 95% CI, 0.99–1.00; *p* = 0.034), male gender (OR, 1.89; 95% CI, 1.60–2.43; *p*<0.001), hypertriglyceridemia (OR, 1.26; 95% CI, 1.09–1.46; *p* = 0.002), hiatus hernia (OR, 21.16; 95% CI, 15.45–28.97; *p*<0.001), current alcohol use (OR, 1.31; 95% CI, 1.12–1.54; *p* = 0.001) and regular exercise (OR, 1.21; 95% CI, 1.01–1.46; *p* = 0.040). In model 2, central obesity was substituted with being obese/overweight, and general obesity (OR, 1.28; 95% CI, 1.07–1.52; *p* = 0.007) and being overweight (OR, 1.25; 95% CI, 1.08–1.45; *p* = 0.003) exhibited a positive association with EE. However, central obesity (OR, 1.06; 95% CI, 0.89–1.26; *p* = 0.549) was not a significant predictor of EE when central obesity and being obese/overweight were simultaneously included in multivariate analysis (model 3).

**Table 2:** The adjusted odds ratio (OR) and 95% confidence interval (CI) of central obesity and overweight/general obesity on the risk of erosive esophagitis based on binomial logistic regression in total population.

Variables	Model 1	Model 2	Model 3
	OR (95% CI)	OR (95% CI)	OR (95% CI)
Age, years	0.99 (0.99∼1.00)*	0.99 (0.99∼1.00)*	0.99 (0.99∼1.00)*
Male vs Female	1.89 (1.60∼2.43)***	1.83 (1.54∼2.17) ***	1.84 (1.55∼2.18)***
Creatinine, µmol/L	0.96 (0.79∼1.16)	0.96 (0.79∼1.17)	0.96 (0.80∼1.17)
Uric acid, µmol/L	1.01 (0.97∼1.07)	1.01 (0.96∼1.06)	1.01 (0.96∼1.06)
Central obesity	1.17 (1.02∼1.34)*		1.06 (0.89∼1.26)
BMI, kg/m^2^			
24∼27 vs <24		1.25 (1.08∼1.45)**	1.22 (1.04∼1.44)*
>27 vs <24		1.28 (1.07∼1.52)**	1.22 (0.98∼1.53)
Hypertension	0.90 (0.77∼1.06)	0.90 (0.76∼1.06)	0.90 (0.76∼1.06)
Diabetes mellitus	1.09 (0.91∼1.31)	1.09 (0.91∼1.31)	1.09 (0.91∼1.31)
Hypertriglyceridemia	1.26 (1.09∼1.46)**	1.25 (1.08∼1.44)**	1.24 (1.08∼1.44)**
Low HDL-C	1.07 (0.93∼1.22)	1.06 (0.92∼1.21)	1.05 (0.92∼1.21)
Hiatus hernia	21.16 (15.45∼28.97)***	21.07 (15.38∼28.85)***	21.06 (15.37∼28.84)***
Current tea drinking	1.02 (0.90∼1.16)	1.01 (0.89∼1.15)	1.01 (0.89∼1.15)
Current smoking	1.05 (0.89∼1.25)	1.07 (0.90∼1.26)	1.07 (0.90∼1.26)
Current alcohol use	1.31 (1.12∼1.54)**	1.31 (1.12∼1.54)**	1.31 (1.12∼1.54)**
Regular exercise	1.21 (1.01∼1.46)*	1.20 (1.00∼1.45)*	1.21 (1.00∼1.45)*
Year of check-up,			
2004∼2006 vs 2001∼2003	1.05 (0.88∼1.27)	1.06 (0.89∼1.28)	1.06 (0.88∼1.27)
2007∼2009 vs 2001∼2003	1.16 (0.96∼1.40)	1.17 (0.96∼1.41)	1.16 (0.96∼1.40)

*p* value *<0.05; **<0.01; ***<0.001.

BMI: body mass index; HDL-C: high density lipoprotein-cholesterol.

As for non-obese group (Table 3), model 1 shows that central obesity (OR, 1.19; 95% CI, 1.00–1.40; *p* = 0.046), male gender (OR, 1.98; 95% CI, 1.64–2.40; *p*<0.001), hypertriglyceridemia (OR, 1.20; 95% CI, 1.01–1.42; *p* = 0.036), hiatus hernia (OR, 19.10; 95% CI, 13.45–27.13; *p*<0.001) and current alcohol use (OR, 1.24; 95% CI, 1.03–1.49; *p* = 0.025) were associated with an increased risk of EE (Table 2). In model 2, we substituted overweight for central obesity in the multivariate analyses, and the result revealed that being overweight (OR, 1.24; 95% CI, 1.07–1.44; *p* = 0.005) was positively related to EE. In model 3, overweight (OR, 1.20; 95% CI, 1.02–1.42; *p* = 0.003) remained as an independent associated factor of EE but central obesity (OR, 1.08; 95% CI, 0.90–1.31; *p* = 0.398) did not. We further analyzed the associated factors of EE in obese group (data not shown) and the result showed age (OR, 0.98; 95% CI, 0.97–0.99; *p* = 0.003), hypertriglyceridemia (OR, 1.42; 95% CI, 1.07–1.87, *p* = 0.015), hiatus hernia (OR, 32.42; 95% CI, 15.64–67.20), *p*<0.001), current alcohol use (OR, 1.57; 95% CI, 1.15–2.15, *p* = 0.004) and regular exercise (OR, 1.53; 95% CI, 1.05–2.22, *p* = 0.027) were positively associated with EE, but central obesity (OR, 0.79; 95% CI, 0.49–1.29; *p* = 0.353) was not.

**Table 3:** The adjusted odds ratio (OR) and 95% confidence interval (CI) of central obesity and overweight on the risk of erosive esophagitis based on binomial logistic regression in non-obese group.

Variables	Model 1	Model 2	Model 3
	OR (95% CI)	OR (95% CI)	OR (95% CI)
Age, years	1.00 (0.99∼1.00)	1.00 (0.99∼1.00)	1.00 (0.99∼1.00)
Male vs Female	1.98(1.64∼2.40)***	1.90 (1.57∼2.30)***	1.92 (1.59∼2.33)***
Creatinine, µmol/L	0.96 (0.79∼1.18)	0.97 (0.79∼1.18)	0.97 (0.80∼1.18)
Uric acid, µmol/L	1.02 (0.97∼1.08)	1.02 (0.96∼1.08)	1.01 (0.96∼1.07)
Central obesity	1.19 (1.00∼1.40)*		1.08 (0.90∼1.31)
BMI, 24∼27 vs <24 kg/m^2^		1.24 (1.07∼1.44)**	1.20 (1.02∼1.42)**
Hypertension	0.83 (0.68∼1.01)	0.83 (0.68∼1.01)	0.82 (0.67∼1.00)
Diabetes mellitus	1.12 (0.90∼1.40)	1.13 (0.91∼1.41)	1.12 (0.90∼1.40)
Hypertriglyceridemia	1.20 (1.01∼1.42)*	1.19 (1.00∼1.40)	1.18 (1.00∼1.40)
Low HDL-C	1.06 (0.91∼1.24)	1.06 (0.91∼1.24)	1.05 (0.90∼1.23)
Hiatus hernia	19.10 (13.45∼27.13)***	19.00 (13.38∼26.99)***	19.00 (13.38∼26.99)***
Current tea drinking	1.04 (0.90∼1.20)	1.03 (0.89∼1.19)	1.03 (0.89∼1.19)
Current smoking	1.09 (0.89∼1.32)	1.10 (0.91∼1.34)	1.10 (0.91∼1.33)
Current alcohol use	1.24 (1.03∼1.49)*	1.24 (1.03∼1.50) *	1.24 (1.03∼1.49) *
Regular exercise	1.13 (0.91∼1.40)	1.12 (0.91∼1.39)	1.12 (0.91∼1.39)
Year of check-up,			
2004∼2006 vs 2001∼2003	1.05 (0.85∼1.29)	1.06 (0.86∼1.31)	1.06 (0.86∼1.30)
2007∼2009 vs 2001∼2003	1.10 (0.89∼1.36)	1.11 (0.89∼1.38)	1.10 (0.89∼1.37)

*p* value: *<0.05; **<0.01; ***<0.001.

BMI: body mass index; HDL-C: high density lipoprotein-cholesterol.

## Discussion

Obesity is widely considered to play an important role in the development of EE [7]–[10], [20] in populations with a mix of different BMI statuses, including normal weight, overweight and obesity. To the best of our knowledge, this is the first study which demonstrates the association of EE with central obesity and being overweight in non-obese subjects (BMI<27). According to our results, when considering the separate effect of central obesity and being overweight (24≤ BMI<27) on EE, both were important associated factors for EE in the non-obese subjects. As the joint effect of being overweight and central obesity on EE, overweight was more detrimental than central obesity in this non-obese population. The result was similar to two previous studies of males with mixed BMI status, not confined to non-obese subjects, which found that BMI exhibited higher risk of EE than WC [21], [22]. With regards to the total population of our study, being overweight (n = 2,414, OR, 1.22, *p* = 0.016) was positively associated with EE but general obesity (n = 1,526, OR, 1.22, *p* = 0.082) showed marginal effect, and this probably due to discrepancy of subgroup sample size since the ORs of being overweight and general obesity were the same from the result of Table 2–model 3 and general obesity (OR = 1.28, *p* = 0.007) exposed a higher OR than being overweight (OR = 1.25, *p* = 0.003) without considering the joint effect of central obesity (Table 2–model 2).

The possible pathogenetic mechanisms of central obesity related esophagitis have been seen as a sequence of processes in recent years [23]. The first of these includes the direct mechanical effects of abdominal obesity, resulting in an elevation of the intragastric and gastroesophageal pressure gradient [10], [24], an increase in acid reflux episodes with longer duration [25], and in transient lower esophageal sphincter (LES) relaxation [26], leading to esophageal mucosal local injury. The second process, from mediator-driven view, is based on inflammatory signals that originate from the visceral adipose tissue, including an increase in proinflammatory cytokines, such as interleukin-6 and tumor necrosis factor-α [27], [28] and a decrease in potentially anti-inflammatory adiponectin [29], [30]. Furthermore, one study showed that adipokines, such as leptin, are positively correlated with WC in overweight subjects, while adiponectin is negatively correlated with it [31]. The resulting humoral changes give rise to systemic low-grade inflammation that might interact with or even enhance the extent of inflammation, such as esophagitis [23], [30], [32], [33].

As for the association between being overweight and EE, the most general plausible mechanism is assumed to be that being overweight may predispose subjects to hiatus hernia, which disrupts the normal anti-reflux mechanisms [34], [35]. Our current study revealed a positive association between being overweight and EE after controlling for hiatus hernia, and thus mechanisms other than hiatus hernia should be considered. Recently, some studies demonstrated an elevation of inflammatory cells or markers in overweight individuals, such as neutrophil-to-lymphocyte ratio, high-sensitivity CRP, chemokines CXCL8 [36] and C-reactive protein [37]. The correlation between EE and inflammatory mediators can perhaps explain the positive association between being overweight and EE in non-obese subjects who may display metabolically obese characteristics, and the cytokines released from the adipose tissue of such individuals resemble those of subjects with obese body figures [31], [38].

Previous studies showed that hiatus hernia is a major associated factor of EE [11], [35] consistent with the present study. The explanation for this is based on the impairment of the clearance of acid exposed at the lower esophagus, backward flow of trapped acid into the esophagus, and weakening of the crural diaphragm as a functional sphincter [39]. The current study revealed a marginal association between hypertriglyceridemia and EE in non-obese subjects. The literature has inconsistent findings with regard to the relationship between hypertriglyceridemia and EE. Some studies found that elevated serum TG is a predictive factor for EE [40]–[42], but others did not [43], [44]. This discrepancy may be due to not adjusting for confounders of EE, such as *Helicobacter pylori* infection and high-fat meals, or different study designs or different study populations. The positive association between hypertriglyceridemia and EE may be due to the fact that hypertriglyceridemia alters the LES pressure or affects esophageal clearance of refluxate [40], or because increased IL-6 concentration stimulates hepatic triglyceride secretion [45] and reduces esophageal circular muscle contraction [46].

In this study, being male was positively associated with EE after adjusting for other risk factors. According to previous studies, males are more prone to have EE [43], [44], [47], [48] while symptomatic the incidence of GERD is similar in males and females [49]. This is probably because males are more likely to have asymptomatic EE [44], [50] and severe reflux disease [2], [51], and the reasons for this include the fact that men are at higher risk of being overweight or obese [47], are more likely to have poor lifestyles (more smoking and greater alcohol consumption) [44], [47], have a higher prevalence of hiatus hernia [47], and a greater parietal cell mass than women [44], [52]. As for the relationship between DM and EE, previous studies revealed that the former was not associated with the latter [44], [53], as also seen in our results. There have been inconsistent results in the literature on the relationship between HTN and EE [41], [43], [44], [54]. In the current study, HTN was not related to EE, as seen in a number of previous works [41], [44]. In contrast, some other studies revealed a positive association between HTN and EE, but they did not adjust for hiatus hernia and some lifestyle factors [43], [54], such as exercise and alcohol and tea drinking.

Finally, the roles of lifestyle risk factors in EE, including alcohol use, smoking, tea consumption and regular exercise, are less well defined. Our study revealed that regular exercise was positively associated with EE in total population, and available evidence indicates that a positive association is present between vigorous exercise and GERD [55], [56], yet study about vigorous exercise on EE is unavailable. Alcohol use was positively associated with EE in this study, as in several earlier works [40], [42], [51], [57]–[59]. However, some studies found no significant association between alcohol use and EE, which may be related to the inclusion of subjects taking GERD medication [32], [41], [44], [47], [60]. Alcohol not only directly damages to the esophageal mucosa by facilitating hydrogen ion penetration [61], but also reduces the LES tone and slows both esophageal motility and gastric emptying [57]. Our study did not find a relationship between smoking and EE in multivariate analysis, and this is consistent with other works [47], [48]. Cross-sectional or case control studies disagree in their conclusions about the effects of smoking on EE [40], [42], [44], [51], [59]. Most studies that examine the risk factors of EE show no significant association with tea drinking [41], [44], [47], similar as in the current work.

There are several limitations to the current study. First, the cross-sectional design cannot be used to establish any cause-and-effect relationship between central obesity/being overweight and EE. Further prospective study is needed to clarify the causal relationship between them. Second, blood samples of various cytokines or adipokines were not obtained for further analysis, so the detailed interactions between systemic inflammatory status and EE in a non-obese population could not be fully explored. Third, no information about reflux symptoms or ambulatory pH monitoring were obtained, and gastric *Helicobacter pylori* infection was not evaluated in the present study. However, the effects of *H pylori* infection or the influence of *H pylori* eradication on EE await further investigation. In this study, the measures of visceral adipose tissue (VAT) were not available, and we could not eliminate the possibly confounding effect of VAT on the relationship between BMI status/central obesity and EE. Finally, some bias could not eliminate from our study design. Since the lifestyle habits and medication were collected from retrospective questionnaire, not by objective means, recall bias might exist. However, we identified current lifestyle habits in recent half year and current medication, so the effect of recall bias might be minimal. Because all enrolled subjects had undergone self-paid health examinations and their socioeconomic status was probably better than the general population, therefore these might lead to a potential selection bias.

In conclusion, overweight effect on EE was more detrimental than central obesity in non-obese subjects. In addition, male gender, hiatus hernia, and alcohol use were also associated with increased risk of EE.
